# Alloying and Doping Control in the Layered Metal Phosphide Thermoelectric CaCuP

**DOI:** 10.1021/acsaelm.3c00828

**Published:** 2023-09-14

**Authors:** Robert J. Quinn, Rajan Biswas, Jan-Willem G. Bos

**Affiliations:** † Institute of Chemical Sciences, School of Engineering and Physical Sciences, Heriot-Watt University, Edinburgh EH14 4AS, U.K.; ‡ EaStCHEM School of Chemistry, University of St Andrews, North Haugh, St Andrews KY16 9ST, U.K.

**Keywords:** metal phosphide, thermoelectric material, alloying, phase boundary mapping, layered semiconductor, ZrBeSi structure

## Abstract

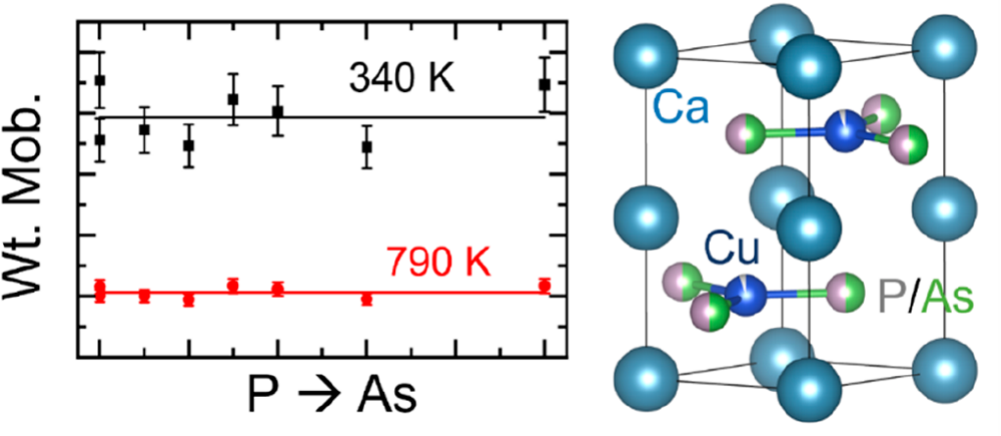

We recently identified CaCuP as a potential low cost, low density thermoelectric material, achieving *zT* = 0.5 at 792 K. Its performance is limited by a large lattice thermal conductivity, *κ*_*L*_, and by intrinsically large p-type doping levels. In this paper, we address the thermal and electronic tunability of CaCuP. Isovalent alloying with As is possible over the full solid solution range in the CaCuP_1–*x*_As_*x*_ series. This leads to a reduction in *κ*_*L*_ due to mass fluctuations but also to a detrimental increase in p-type doping due to increasing Cu vacancies, which prevents *zT* improvement. Phase boundary mapping, exploiting small deviations from 1:1:1 stoichiometry, was used to explore doping tunability, finding increasing p-type doping to be much easier than decreasing the doping level. Calculation of the Lorenz number within the single parabolic band approximation leads to an unrealistic low *κ*_*L*_ for highly doped samples consistent with the multiband behavior in these materials. Overall, CaCuP and slightly Cu-enriched CaCu_1.02_P yield the best performance, with *zT* approaching 0.6 at 873 K.

## Introduction

**Figure 1:**
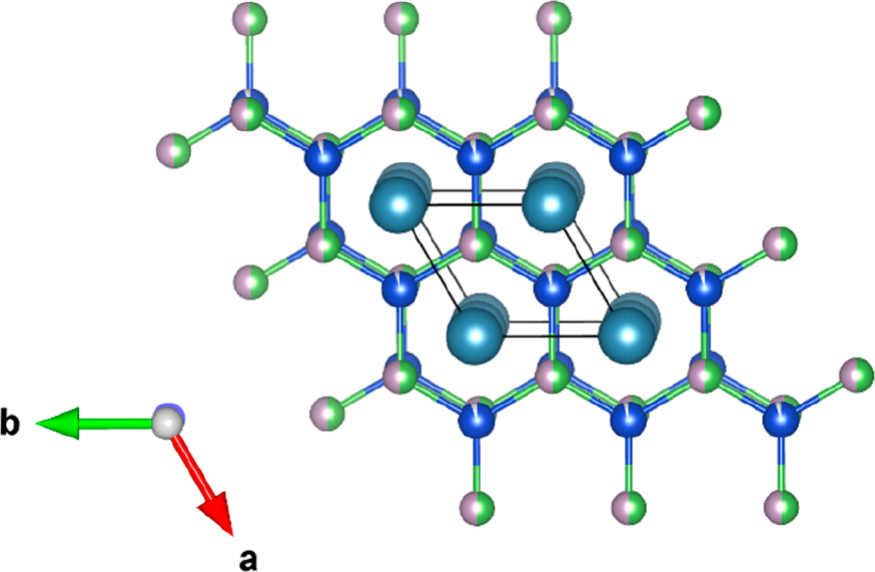
Schematic representation of the layered CaCuP structure, viewed down the hexagonal *c*-axis, showing P/As (green/gray atoms) alloying in the hexagonal boron nitride layers and the occurrence of Cu (blue atoms) vacancies. (Ca atoms are metallic blue.)

Thermoelectric materials convert between a thermal gradient and electricity and can be used in power generation, scavenging, and thermal control applications.^1−3^ The past two decades has seen rapid advances in performance in a wide range of thermoelectric materials, driven by new design concepts.^4−9^ Metal phosphides are not yet well explored but have recently emerged as a promising class of thermoelectric materials.^10,11^ Structural complexity supports low thermal conductivities, despite a low average atomic mass, while the variable bonding of phosphorus affords opportunities to optimize the power factor.^10^ The efficiency of a thermoelectric material is given by its figure of merit, *zT* = (*S*^2^/ρκ)*T*. Here, *S* is the Seebeck coefficient, ρ the electrical resistivity, and κ the sum of lattice (*κ*_*L*_) and electronic (*κ*_*E*_) thermal conductivities. The current best metal phosphide thermoelectrics are n-type Cd_3_P_2_ with *zT* approaching 1,^12,13^ while *zT* = 0.5–0.7 is achieved in a range of p-type materials, including tetrahedrite Ag_6_Ge_10_P_12_,^14−17^ clathrates,^18,19^ 122-type AZnCuP_2_ (A = Ca, Eu, Yb),^20,21^ and 111-type CaAgP and CaCuP.^22,23^ The latter stands out due to its good electronic properties with the largest observed power factors (*S*^2^/ρ) in this class of materials.^23^ The good electronic properties are related to the presence of two highly dispersive valence bands associated with in-plane electrical transport.^24^ From DFT calculations, these bands have very low band masses, *m*_b_* = 0.2 m_e_ and *m*_b_* = 0.4 m_e_.^23,24^ These afford large hole mobilities, *μ*_*H*_ ≈ 100 cm^2^ V^–1^ s^–1^ at 300 K in polycrystalline samples.^23^ A recent paper on isostructural BaAgSb achieved *μ*_*H*_ ≈ 350 cm^2^ V^–1^ s^–1^ at 300 K.^25^ This is one of the largest values observed in Zintl thermoelectrics,^26^ with this report linking these high mobilities to the crystal symmetry,^25^ suggesting they are an intrinsic feature of the ZrBeSi structure type. The crystal structure of CaCuP is illustrated in Figure 1 and consists of hexagonal BN-like CuP layers interspersed by layers of Ca metal.

CaCuP has recently attracted some interest as a prospective transparent optical conductor with good mobilities, *μ*_*H*_, ∼45 cm^2^ V^–1^ s^–1^ at 300 K, in polycrystalline thin films.^24,27^ MgCuP was part of our previous study and also has *zT* ≈ 0.5 at 800 K, but this is based on a lower *κ*_*L*_ and moderate *S*^2^/ρ, reflecting its different crystal structure.^23^ In addition, MgCuP shows evidence of a reversible high-temperature decomposition that is absent for CaCuP, which has better stability.^23^ SrCuP is isostructural to CaCuP and was recently reported with *μ*_*H*_ ≈ 30 cm^2^ V^–1^ s^–1^ at 300 K and *zT* ≈ 0.2 at 600 K, with room for improvement if sample quality can be improved.^28^ Dilute ferromagnetic EuCuP compositions are investigated as magnetic topological materials and appear too metallic for thermoelectric applications.^29−31^

Metal phosphides are typically characterized by low gravimetric densities, compared to many of the state-of-the-art thermoelectric materials,^23^ which could be advantageous for portable applications. They also possess good high-temperature stability in many cases, although care has to be taken with respect to release of PH_3_(g), which can evolve upon reaction with moisture, and sublimation of P_4_(g) over extended periods of time at elevated temperatures.^10^ A good description of the necessary experimental precautions can be found in the initial work on phosphide tetrahedrite materials.^14^

Here, we report an optimization study of the promising metal phosphide CaCuP. Isovalent alloying with As has been successfully used to reduce *κ*_*L*_, while growth from off-stoichiometric compositions was attempted to suppress high levels of intrinsic p-type doping, attributed to the facile formation of Cu vacancy defects.^23,27^ In the Zintl limit, Ca and Cu donate electrons into P states, achieving Ca^2+^, Cu^+^, and P^3–^ closed-shell electronic configurations. Any shortage of Cu (or Ca), will therefore lead to a shortage of electrons and unfilled P-states, leading to p-type conduction. Unfortunately, the favorable suppression of *κ*_*L*_ in the alloyed compositions is undone by higher levels of intrinsic p-type doping, leading to compromised *S*^2^/ρ and no overall improvement in *zT*. Interestingly, the electronic quality of the CaCuP_1–*x*_As_*x*_ solid solution, given by the weighted electronic mobility, does not show an alloying suppression and remains high for all *x*. Nominally stoichiometric CaCuP and slightly Cu-rich CaCu_1.02_P compositions are closest to the possible optimal *S*^2^σ but remain overdoped with a large *κ*_*E*_ compromising *zT*.

## Experimental Section

All precursors and samples were handled in an argon atmosphere glovebox. The hot-pressed disks were found to be stable on the bench over a period of weeks at least. Samples were prepared from the following elemental precursors: Ca granules (Alfa Aesar, 99.5%), Cu powder (Alfa Aesar, 99.9%), P lump (Alfa Aesar, 99.999%), and As lump (Sigma-Aldrich, 99.999%). Cu, P, and As were ground together using a mortar and pestle for 20 min and then transferred to a carbonized silica ampule with Ca granules, in the appropriate stoichiometric ratio. The ampules were then sealed under vacuum using a blowtorch. The ampules were then heated in a box-furnace, ramped up at 2 °C min^–1^, held at 200 °C for 3 h, then 600 °C for 3 h, then 900 °C for 2 h, at which point the ampules were cooled at a rate of 3 °C min^–1^ to room temperature. This sequence was chosen out of caution to prevent large P vapor pressure building up inside the sealed tubes. Samples were then ground into a powder, cold-pressed into 13 mm pellets at an applied pressure of 5 tons, and resealed in evacuated quartz ampules. The ampules were heated again in a box-furnace, using the same ramp rates but now left at 900 °C for 24 h. The resulting samples were reground once more into powder. 0.9–1 g of material was then hot-pressed into 13 mm pellets using a home-built instrument using graphite dies and induction heating. Samples were pressed with a maximum pressure of ∼80 MPa and temperature of 950 °C, yielding dense samples (Table 1), confirmed by the geometric method. After hot-pressing, the samples were annealed at 900 °C inside vacuum sealed ampules using the previous heating profile. No mass loss or condensation of white P was observed during the synthetic protocol.

**Table 1:** Overview of Fitted Lattice Parameters, Unit Cell Volume, *c*/*a*-Ratio, and Gravimetric Densities for the CaCuP_1–*x*_As_*x*_ Solid Solution and the CaCuP Samples Prepared as Part of the Phase Boundary Mapping Study

	*a* (Å)	*c* (Å)	vol (Å^3^)	*c*/*a*	density (g cm^–3^)
**CaCuP**_**1–*x***_**As**_***x***_**solid solution**					
0	4.0560(1)	7.8050(1)	111.20(1)	1.924	3.75
0.1	4.0719(1)	7.8151(1)	112.22(1)	1.919	4.07
0.2	4.0853(1)	7.8224(1)	113.06(1)	1.915	4.06
0.3	4.0987(1)	7.8307(1)	113.93(1)	1.911	4.34
0.4	4.1121(1)	7.8382(1)	114.78(1)	1.906	4.33
0.6	4.1373(1)	7.8512(1)	116.39(1)	1.898	4.45
1	4.1878(1)	7.8720(1)	119.56(1)	1.880	4.72
**phase boundary mapping**					
Ca_1.05_CuP	4.0557(1)	7.8016(1)	111.13(1)	1.924	3.76
Ca_0.95_CuP	4.0556(1)	7.8030(2)	111.15(1)	1.924	3.89
CaCu_1.05_P	4.0558(1)	7.8044(1)	111.18(1)	1.924	3.90
CaCu_0.95_P	4.0545(1)	7.7951(2)	110.98(1)	1.922	3.94
CaCu_0.93_P	4.0549(1)	7.7978(2)	111.04(1)	1.923	3.87
CaCuP_1.05_	4.0555(1)	7.8008(2)	111.10(1)	1.924	3.97
CaCuP_0.95_	4.0562(1)	7.8049(1)	111.20(1)	1.924	3.80

X-ray powder diffraction (XRD) data were collected on all prepared samples using a Malvern Panalytical Empyrean diffractometer in Bragg–Brentano geometry using a nonmonochromated CuK_α_ beam. Rietveld analysis was performed using Topas V6 software with jEdit used to write input files.^32^ In order to identify the secondary phases present in the XRD data, scanning electron microscopy (SEM) and energy dispersive X-ray spectroscopy (EDX) measurements were performed on a JEOL JSM-IT200 instrument equipped with a JEOL DrySD EDX spectrometer and operated at 20 kV.

Thermal diffusivity (α) values were collected on the hot-pressed disks between 300–800 K using a Linseis LFA-1000 laser flash apparatus with a graphite coating to reduce emissivity errors. Following diffusivity measurement, bars were cut from the disk (8–10 mm in length) for electrical property measurements. *S*(*T*) and ρ(*T*) were measured using a Linseis LSR-3 apparatus with a four-probe setup. Dulong-Petit values were used for the heat capacity in the calculation of the thermal conductivity.

## Results

### CaCuP_1–*x*_As_*x*_

**Figure 2:**
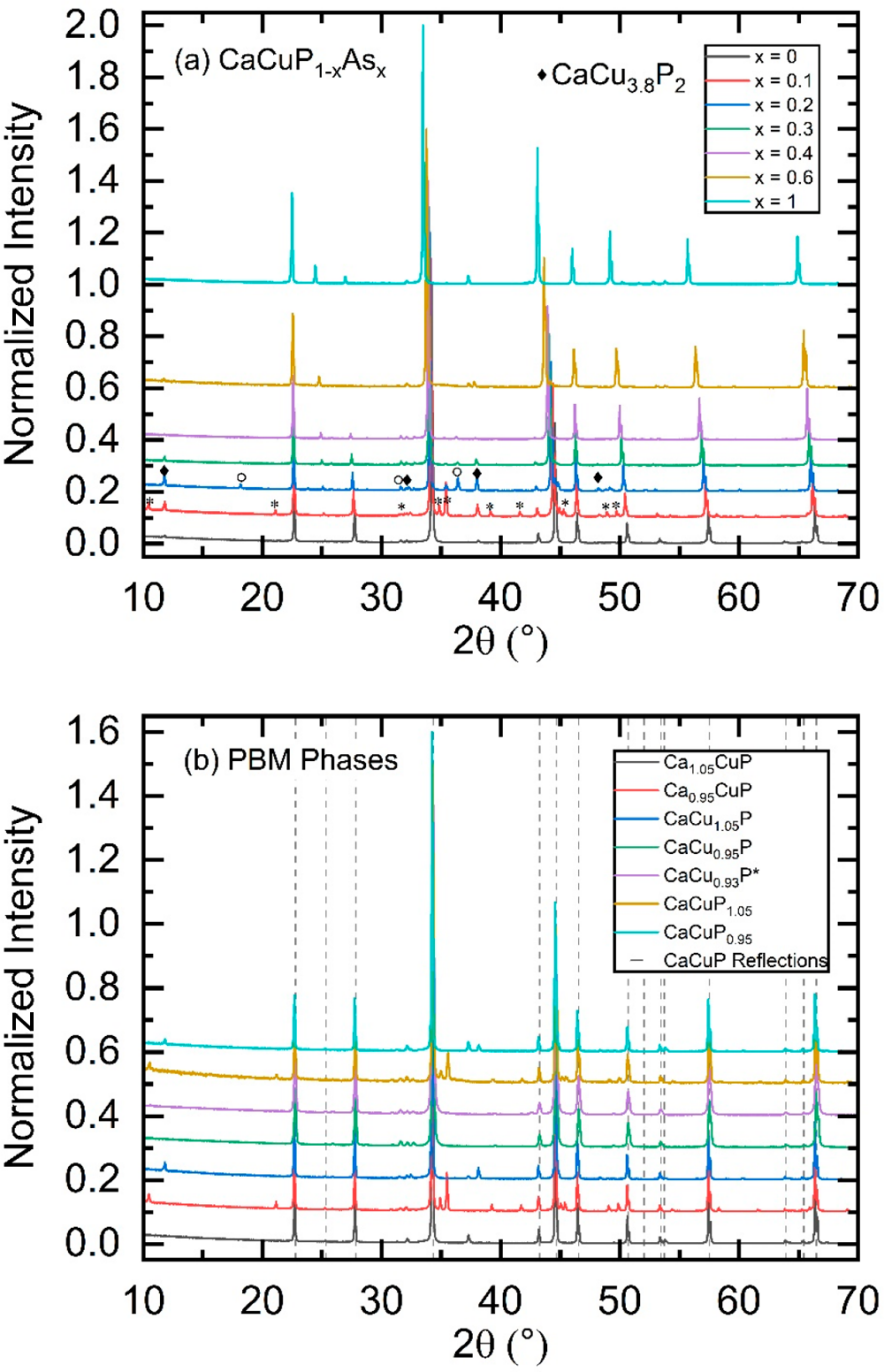
Stacked plots of XRD data collected on (a) the CaCuP_1–*x*_As_*x*_ solid solution and (b) the off-stoichiometric CaCuP samples in the phase boundary mapping study. Starred (*) and open circle (ο) impurities remain unidentified and occur in varying amounts in data collected from different parts of the same sample, as shown more fully in the Supporting Information.

XRD patterns for all of the prepared samples are shown in Figure 2a. The end-members were found to be nearly phase pure, but some of the intermediate compositions contained impurity phases. These proved very difficult to index using any of the known Ca–Cu–P–As phases, with only trigonal CaCu_3.8_P_2_ unambiguously identified. Furthermore, data collection on different parts of the same sample yielded different intensities for the most intense impurities (Figure S1 in the Supporting Information). This enabled the reflections belonging to the same impurity to be identified, marked with (*) and (ο) in Figure 2a. However, it proved impossible to identify the impurity phases by matching against the crystallography open database, even when allowing for oxide, carbide, or silicide formation. SEM-EDX was performed on selected samples (Figures S3–S4; Table S1). This revealed the presence of two Ca–Cu–P–As impurities. The most obvious corresponds to CaCu_3.8_P_2_, also observed in XRD, and the other more subtle phase is a another Cu-rich composition with approximate M_2_P composition, where M is a mixture of Ca–Cu. In addition, in localized areas, increased levels of oxygen were observed, coupled to the presence of Ca–Cu–P, suggesting the formation of a Ca–Cu phosphate phase. The observation of a localized impurity is consistent with the variation in X-ray intensities for measurements on the same sample. The observation of two additional impurity phases in SEM-EDX is consistent with the presence of the (*) and (ο) phases from XRD. However, we have been unable to link these to a crystallographic structure.

**Figure 3:**
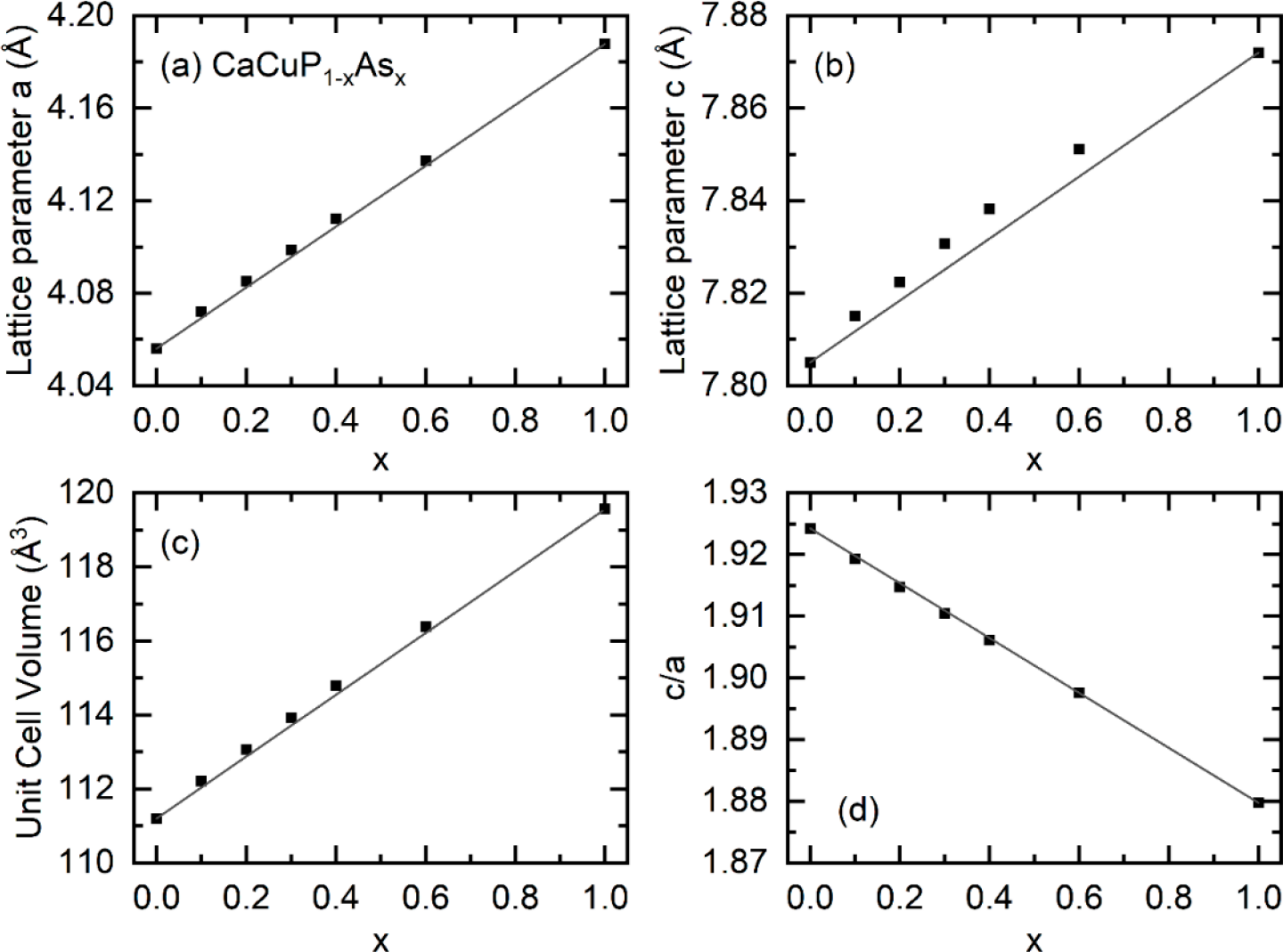
Lattice parameters in the CaCuP_1–*x*_As_*x*_ solid solution determined from Rietveld refinement of XRD data. (a) shows the in-plane lattice parameter *a*, (b) shows the cross-plane lattice parameter *c*, (c) shows the unit cell volume, and (d) shows the *c*/*a* ratio, demonstrating faster expansion in-plane compared to the spacing between the CuP_1–*x*_As_*x*_ layers.

The reflections of the main CaCuP phase show a gradual shift in peak positions and remain sharp throughout, confirming the successful substitution of As on the P site in the crystal structure. The refined lattice parameters of the CaCuP_1–*x*_As_*x*_ solid solution are shown in Figure 3 and are listed in Table 1. The substitution of As into the structure leads to steady increase in the *a* and *c*, from 4.056 to 4.188 Å (+3.25%) and 7.805 to 7.872 Å (+0.85%), respectively, as shown in Figure 3a,b. The expansion of the *a*-axis and unit cell volume (Figure 3c) is almost linear, while the *c*-axis has a slightly more convex dependence. The near linear expansion of the unit cell metrics confirms the formation of a solid solution. The *x* = 0.6 sample was made with a 5% reduced P content (i.e., an excess of electron donating Ca, Cu), to try to reduce the p-type carrier concentration but is found to follow both structural and thermoelectric trends of the stoichiometric samples. The lattice parameters of the end-members are in good agreement with the original work reporting these phases.^33^ The alloying dependence of the *c*/*a* ratio (Figure 3d) confirms that the in-plane expansion is faster than that in the *c*-direction. The in-plane expansion follows the increase in the Cu–P/As bond length, from 2.34 to 2.42 Å, while the expansion in the *c*-direction is buffered by the presence of the Ca^2+^ cations. Trial refinement of the Cu-site occupancies suggests a small 0–1% deficiency for alloyed samples up to *x* = 0.6, with a larger (3–5%) deficiency for CaCuAs. This suggests an increasing level of Cu deficiency with alloying, which is consistent with the observed change to more metallic behavior in the thermoelectric property data discussed below.

**Figure 4:**
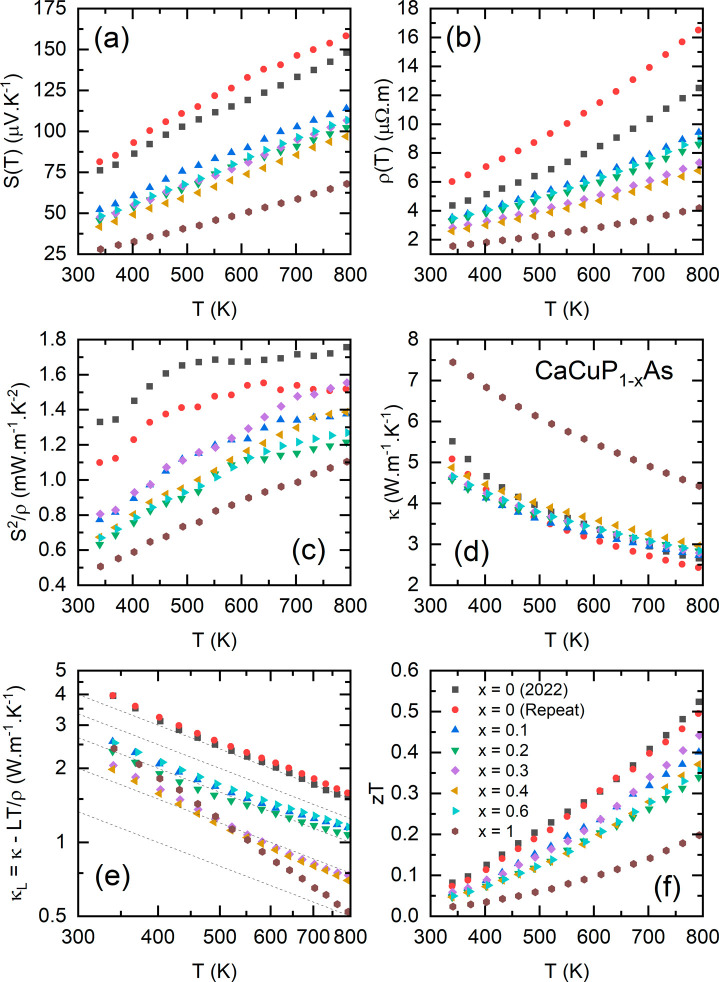
Thermoelectric property data for the CaCuP_1–*x*_As_*x*_ solid solution. (a) shows the absolute Seebeck coefficient *S*(*T*), (b) shows the electrical resistivity ρ(*T*), (c) shows the power factor *S*^2^/ρ, (d) shows the total thermal conductivity κ(*T*), (e) shows κ(*T*) minus the expected electronic thermal conductivity κ_*E*_(*T*), (f) shows the figure of merit *zT*. The dashed lines in (e) are the expected *T*^–1^ temperature dependence for Umklapp phonon scattering, with highly doped CaCuAs showing an unphysical rapid decrease.

The thermoelectric properties of the CaCuP_1–*x*_As_*x*_ series are shown in Figure 4. For each sample, *S*(*T*) is near linear with positive magnitude, consistent with highly doped p-type semiconducting behavior. The *S*_*340 K*_ values for CaCuP of +76 and +81 μV·K^–1^ are substantially larger than those of CaCuAs with +28 μV·K^–1^. Similarly, ρ(*T*) for all samples has a positive temperature dependence, consistent with highly doped degenerate semiconducting behavior, with *ρ*_*340 K*_ values of 4.4 and 6.0 μΩ·m for the two CaCuP samples, being substantially larger than CaCuAs which has 1.55 μΩ·m. These values indicate that CaCuAs is more highly doped than CaCuP, with alloyed samples having ρ(*T*) values intermediate between the end-members.

The CaCuP samples exhibit the largest *S*^2^/ρ across the full temperature range, achieving a broad plateau of 1.6–1.7 mW·m^–1^·K^–2^ from 500 to 792 K. These are among the largest *S*^2^/ρ observed in metal phosphide materials.^10^ The alloyed samples have lower *S*^2^/ρ, which is due to being too highly doped, as will be discussed later on.

In terms of thermal transport, the *κ(T)* values shown in Figure 4d are quite similar for all samples except for CaCuAs, which has *κ*_*340 K*_ ≈ 7 W·m^–1^·K^–1^, compared to values of 4–5 W·m^–1^·K^–1^ for the rest of the series. For the most part, this difference is due to the larger *κ*_*E*_, which is usually calculated by using the Wiedemann–Franz law, where *κ*_*E*_ = *LT*/ρ, where *L* is the Lorenz number. In most cases, *L* can be estimated using an effective Single Parabolic Band model or an empirical equation that gives to within 10% accurate results.^34^ However, in the case of CaCuP, two valence bands contribute to the electronic transport.^23,24^ Each of these individual bands will be less highly doped (compared to a nominal single band) and hence will have a lower *L*, leading to a potential overestimate of *κ*_*E*_ if the SPB approximation is used.^34,35^ The data show that the SPB approximation is indeed not adequate for the materials studied here, and this is particularly evident for highly conducting CaCuAs. Figure 4e shows a plot of κ – *κ*_*E*_ = *κ*_*L*_, which shows an unphysically large reduction with an increasing temperature for CaCuAs. Typically the strongest temperature expected dependence for *κ*_*L*_ is *T*^–1^ for Umklapp scattering,^35^ which is indicated by gray lines in Figure 4e. The temperature dependence for CaCuAs is significantly larger than this behavior. This is evidence that *L* and *κ*_*E*_ are overestimated by using the SPB approximation, which is consistent with the multiband behavior predicted from DFT calculations.^24^ In the less highly doped CaCuP_1–*x*_As_*x*_ samples, any error in *κ*_*E*_ will have a lower impact on the calculation of *κ*_*L*_, but the obtained values will not strictly be correct.

*zT* for the CaCuP_1–*x*_As_*x*_ series is shown in Figure 4f. While there is a reduction in *κ*_*L*_ due to increased point defect scattering, this is offset by the reduced *S*^2^/ρ and large *κ*_*E*_, leaving CaCuP with the highest *zT* value, reaching 0.5 at 792 K.

**Figure 5:**
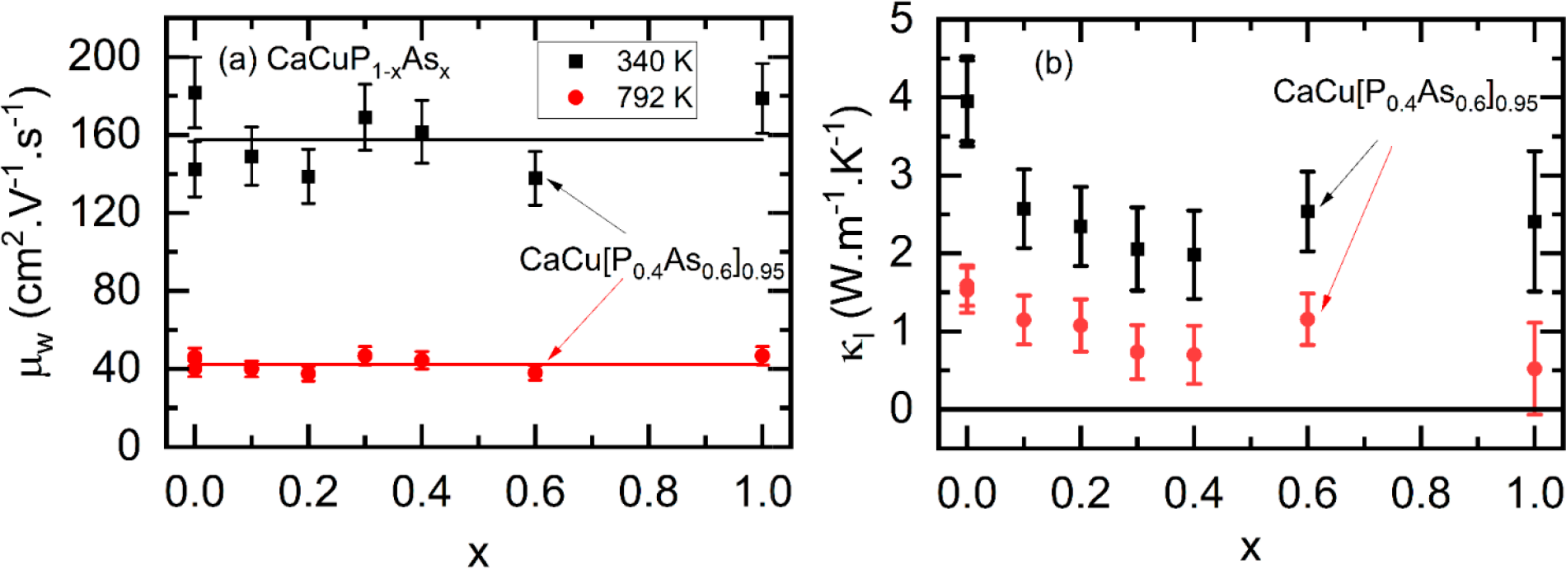
Plots of the effects of alloying on (a) the weighted mobility μ_*w*_ and (b) the lattice thermal conductivity κ_L_ at 340 and 792 K. Error bars in (b) are calculated as the combination of the total and electronic thermal conductivity errors, each estimated to be 10%.

Figure 5 shows the weighted mobility (*μ*_*w*_) and *κ*_*L*_ as functions of alloying composition. Here, *μ*_*w*_ = μ_0_(m*_DoS_/*m*_*e*_*)*^3/2^ is the product of the intrinsic carrier mobility (*μ*_0_) and the density of states effective mass (m*_DoS_) and is a measure of the electronic quality of thermoelectric material.^36^*μ*_*w*_ can be estimated from the measured *S*(*T*) and ρ(*T*) under the assumption of acoustic phonon scattering (APS) dominated charge transport and SPB behavior. This has been shown to be a reasonable approximation for many thermoelectric materials.^36^ However, as discussed above, there is some evidence that CaCuP cannot be fully described using an effective SPB (demonstrated by the overestimation of *κ*_*E*_). In addition, polar optical phonon scattering has been indicated as an important scattering mechanism in some metal phosphide materials.^20^ Nevertheless, even with the SPB and APS approximation, the calculation of *μ*_*w*_ allows insight into important electronic trends. Interestingly the calculated *μ*_*w*_ does not show any alloying dependence and remains invariant at both 340 and 792 K. This contrasts with *κ*_*L*_, which shows a clear Klemens-type alloying reduction,^37^ consistent with the expected impact of mass fluctuations. The unchanged *μ*_*w*_ confirms that the reduced *S*^2^/ρ for the alloyed samples is almost certainly due to being too highly doped and not to a change in band structure (m*_DoS_) or intrinsic carrier mobility (*μ*_0_). It is clear that in order to improve *zT*, control of intrinsic levels of hole doping is required in these materials. As mentioned, the p-type behavior is most likely caused by the formation of Cu vacancies, which in principle can be controlled by altering the chemical potentials of the elements present in the reaction mixture.^38^ For the ternary Ca–Cu–P system, this involves growth from a range of off-stoichiometric compositions. The most systematic approach is to grow CaCuP from all phase fields in the ternary Ca–Cu–P phase diagram, an approach labeled phase boundary mapping (PBM).^38^ Defect energy calculations have been done for CaCuP in the context of transparent conducting films.^27^ These reveal that Cu vacancies are always the lowest energy defect, with an increased formation energy for Cu-rich and P-poor conditions.

### Phase Boundary Mapping

**Figure 6:**
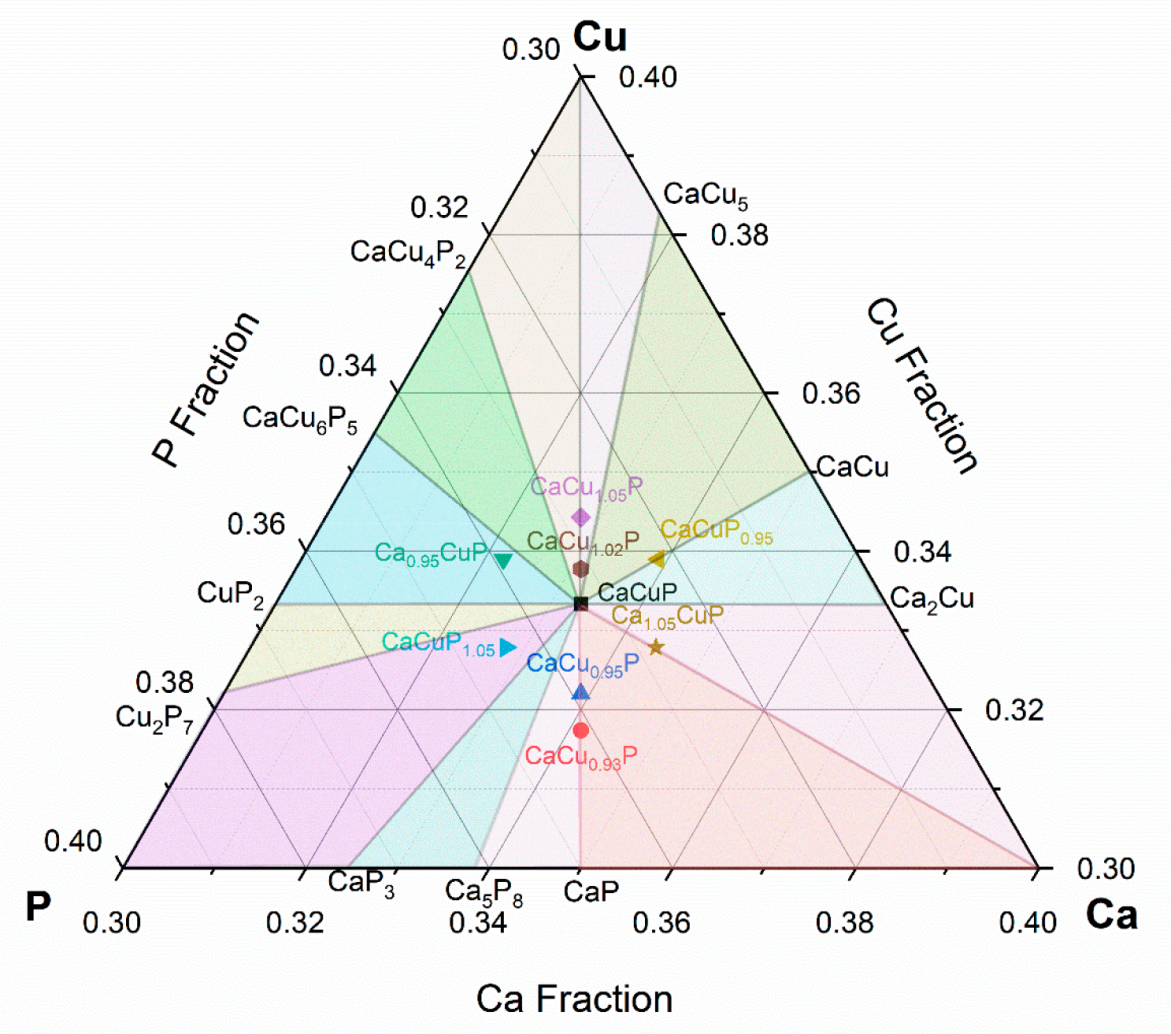
Ternary Ca–Cu–P phase diagram showing the unique phase fields, based on stable compositions from the Materials Project database,^39^ and nominal compositions of the prepared samples.

The phase fields in the ternary Ca–Cu–P phase diagram, based on the Materials Project database,^39^ are shown in Figure 6. As a first attempt, six samples with 0.05 deviation from CaCuP stoichiometry were studied. These correspond to the trivial cases of ±0.05 Ca, Cu, or P and sometimes lie on the boundary between two phase fields, rather than inside. After this initial set of samples, two further compositions, CaCu_1.02_P and CaCu_0.93_P, were prepared. Excess Ca and Cu are linked to n-type doping, and an excess of accepting P will lead to p-type conduction. XRD analysis of the samples produced for PBM analysis did not show any significant differences in the lattice parameters of the CaCuP phase (Table 1 and Figure 2b). The main variation in the XRD patterns was observed in the impurity peaks, with different phases observed depending on nominal composition. As was the case for the As alloying series, the secondary phases proved to be difficult to identify, even using SEM-EDX (Figures S2, S5–7, Table S1). As before, the only unambiguous phase is trigonal CaCu_3.8_P_2_, which is observed for CaCu_1.05_P and CaCuP_0.95_. Based on the phase diagram, the observation of this phase is expected for CaCu_1.05_P but not for CaCuP_0.95_, where Ca–Cu phases should occur (Figure 6). The same unidentified (*) and (ο) impurity phases are also present in these samples, which we believe to be (at least partly) due to localized extrinsic impurities, potentially phosphate based. The observed XRD patterns were matched against all known phases in the Ca–Cu–P phase diagram, but the other remaining small impurity peaks were not identified. In these samples, SEM-EDX also finds evidence for other Ca–Cu–P phases, primarily with approximate M_2_P stoichiometry. This composition is not close to a known phase, suggesting that other as-yet-unknown compounds may be found in the ternary Ca–Cu–P phase diagram.

**Figure 7:**
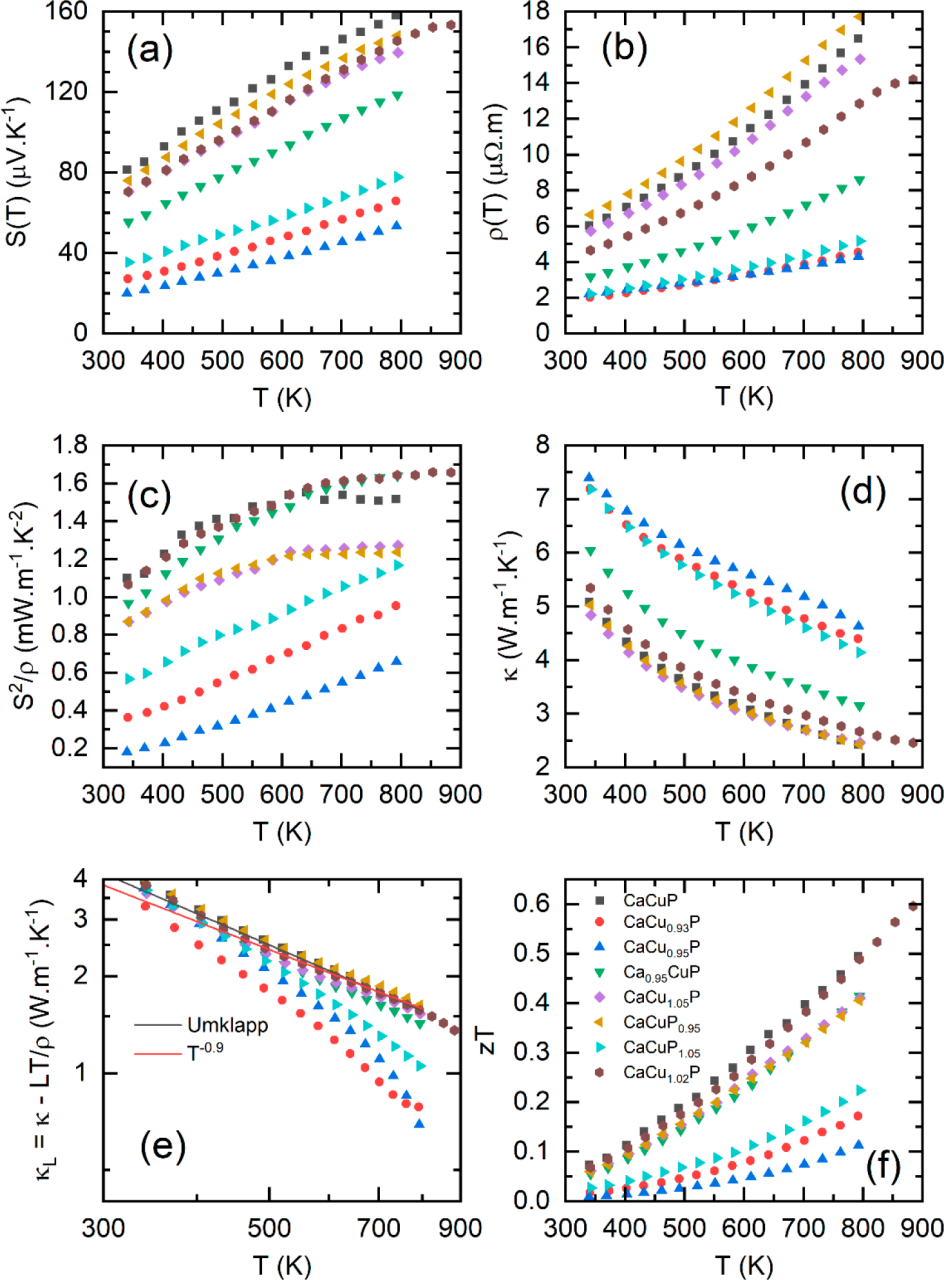
Thermoelectric property data for the CaCuP phase boundary mapping study. (a) shows the absolute Seebeck coefficient *S*(*T*), (b) shows the electrical resistivity ρ(*T*), (c) shows the power factor *S*^2^/ρ, (d) shows the total thermal conductivity κ(*T*), (e) shows κ(*T*) minus the estimated electronic thermal conductivity κ_*E*_(*T*), (f) shows the figure of merit *zT*. Highly doped samples in panel (e) again show a deviation from the expected Umklapp behavior.

The thermoelectric properties of the PBM samples are listed in Figure 7. As was the case for the alloyed samples, all PBM samples show *S*(*T*) and ρ(*T*) consistent with p-type doped degenerate semiconductor behavior. In both *S*(*T*) and ρ(*T*), there is a clear divide between the target p-type samples with low *S*(*T*) and ρ(*T*) and the target n-type samples that are close to CaCuP. Ca_0.95_CuP is intermediate in terms of *S*(*T*) and ρ(*T*), which may indicate that the Ca content has a limited influence on the formation of Cu vacancies, with the Cu/P ratio holding more significance. None of the samples have a larger *S*(*T*) than stoichiometric CaCuP, which is the key indicator for a reduced carrier concentration in a system with fixed electronic structure (*S* ∝ m*_DoS_/*p*^2/3^ in the highly doped limit; were *p* is the hole concentration).^7^ The observation of a slightly larger ρ(*T*) for CaCuP_0.95_ therefore reflects a reduction in carrier (Hall) mobility, which may simply be due to the presence of impurity phases. The measured *S*(*T*) and ρ(*T*) indicate the presence of two sets of samples: The first (“nondoped”) are close to CaCuP in terms of doping level with some reduction in sample quality. The second are highly p-type doped (“overdoped”), leading to strongly compromised performance. This can be seen in the *S*^2^/ρ values shown in Figure 7c. The peak *S*^2^/ρ for “nondoped” CaCuP_0.95_ and CaCu_1.05_P is lower than for CaCuP, reflecting their lower sample quality. The “overdoped” samples have reduced *S*^2^/ρ, with their peak pushed above 800 K. Ca_0.95_CuP has a similar peak *S*^2^/ρ to CaCuP, again suggesting that Ca content is less important than the Cu/P ratio. Synthesis of a CaCu_1.02_P sample yielded similar *S*^2^/ρ values to the CaCuP parent material, showing that small amounts of excess Cu do not reduce sample quality, nor do they reduce the intrinsic p-type doping level.

For the thermal conductivity (Figure 7d), a similar division between “nondoped” and “overdoped” samples occurs, which can be attributed to a larger *κ*_*E*_ for the highly doped samples. The subtraction of *κ*_*E*_(*T*) to give *κ*_*L*_(*T*) is shown in Figure 7e, showing most samples have a very similar temperature dependence, close to *T*^–1^ Umklapp behavior. As for the CaCuP_1–*x*_As_*x*_ series, the most highly doped samples again show a faster than 1/*T* reduction, likely due to an overestimation of *κ*_*E*_. From the *zT* data shown in Figure 7f, it is clear that overdoping leads to a drastic reduction in performance. The CaCu_1.02_P sample has *zT* comparable to that of stoichiometric CaCuP, and measurement up to higher temperature shows a continued increase in *zT*, approaching 0.6 at 873 K.

## Discussion

We have investigated the impact of As alloying in CaCuP and attempted to control the p-type doping level in CaCuP. The main results are that alloying does result in a reduction of *κ*_*L*_ but also leads to increased intrinsic p-type doping that moves *S*^2^/ρ farther from optimal values. The high levels of p-type doping are caused by the presence of Cu vacancies, which increase with As alloying. This behavior may be driven by a reduction in electronic bandgap, lowering the Cu vacancy defect formation energy. Interestingly, the electronic quality (*μ*_*w*_) of CaCuP is unchanged with alloying, indicating that similar *S*^2^/ρ values are possible for all compositions in the CaCuP_1–*x*_As_*x*_ solid solution. However, the occurrence of intrinsically high and increasing levels of p-type doping as *x* increases prevents optimal values of *S*^2^/ρ to be achieved. The result of our PBM study is in keeping with that obtained on CaCuP thin films grown by magnetron sputtering.^27^ That study was focused on increasing σ, which is favored by low Ca/Cu over P ratios, consistent with introducing additional Cu acceptor vacancies.

**Figure 8:**
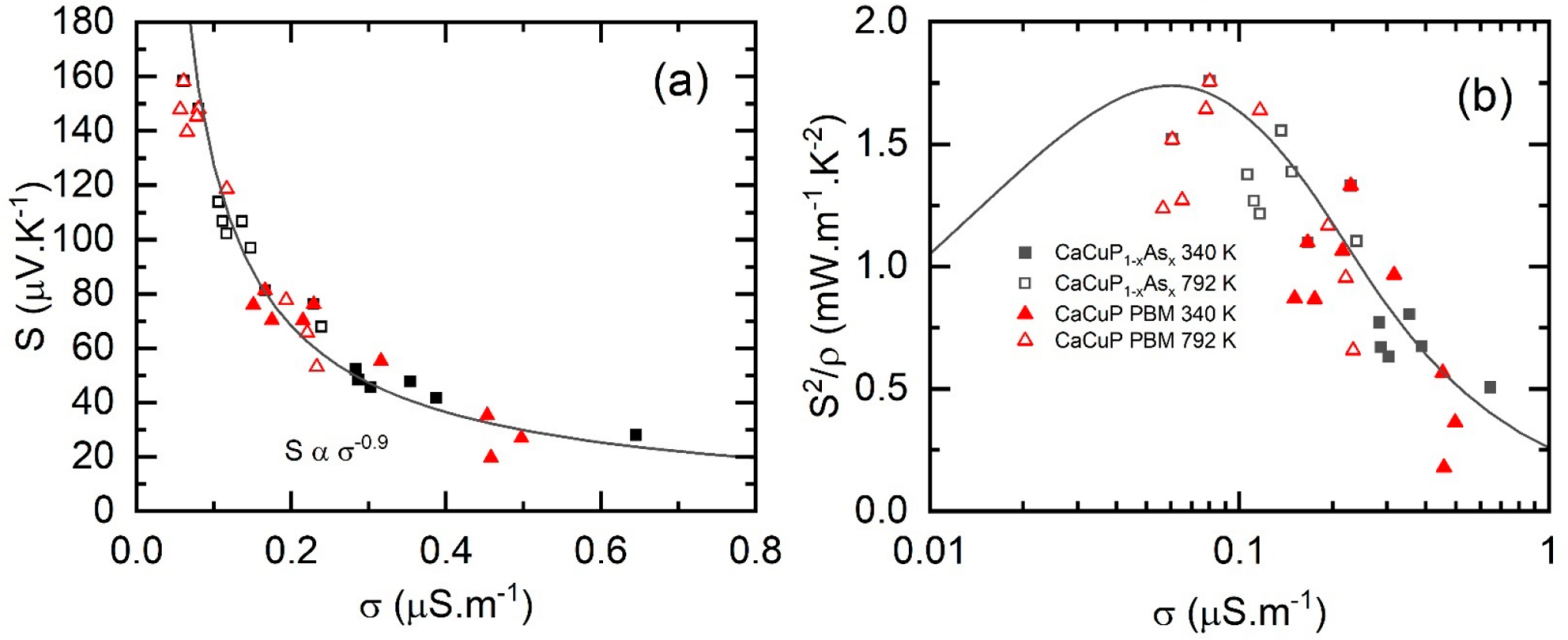
(a) Jonker-Ioffe plot of the Seebeck coefficient (*S*) against electrical conductivity (σ) at 340 and 792 K for all samples in this work. Most data points lie on a universal curve, highlighting the similar thermoelectric behavior. (b) Plot of the power factor *S*^2^/ρ vs σ for all samples in this work. At 340 K, all samples are significantly overdoped. At 792 K, samples begin to approach optimal doping. Note that *zT* is optimized at lower σ, where κ_*E*_ is smaller. The empirical equation for μ_*w*_^36^ is rearranged with σ written in terms of *S*, *T*, and μ_*w*_. At fixed μ_*w*_ and *T*, σ values are calculated for a range of input *S* values, allowing the expected *S*^2^σ dependence on σ to be calculated. Using this model, the calculated *S*^2^σ–σ trend is the same at all temperatures.

A Jonker-Ioffe plot^40^ of *S* against σ at 340 and 792 K for all alloyed and PBM samples is shown in Figure 8a. All data points fall on a single line, indicating a largely unchanged m*_DoS_ and carrier (Hall) mobility across all samples. This again shows that substituting P with As does not lead to substantial changes to the electronic properties. The solid line shows a predicted trend line based on *S*(*T*) ∼ *T*^1^ and ρ(*T*) ∼ *T*^1.9^ with only the least doped samples deviating substantially due to the nonlinearly of *S*(*T*). The exponent for ρ(*T*) comes from fitting ρ(*T*) = *ρ*_0_ + *BT*^*n*^,^41^ where *n* = 1.9 roughly fits the majority of the samples. Here, *n* = 1.9 is purely empirical and is larger than *n* = 1.5 predicted for transport dominated by acoustic phonon scattering.^41^ This result is comparable to the fitting of μ(*T*) for CaCuP thin films (5–300 K), which has a similar temperature dependence (*n* = 2.05 converted to our ρ(*T*) notation).^27^

Figure 8b plots *S*^2^/ρ against σ for all samples, along with expected behavior determined using the empirical equation for *μ*_*w*_.^36^ All data are located on the right-hand side of the curve, which in terms of maximizing *S*^2^/ρ is the overdoped region, in particular, 340 K. At the highest measured temperatures, the best samples approach the optimum *S*^2^/ρ. However, it is worth pointing out that in terms of maximizing *zT*, the optimal doping level lies further toward the left of the *S*^2^/ρ maximum. A hypothetical alloyed sample, where doping can be controlled and *S*^2^/ρ optimized, can achieve *zT* ≈ 1 at 873 K. A trial attempt to use La substitution on the Ca site, as a compensating n-type dopant, did not succeed in lowering the carrier concentration and did not improve *S*^2^/ρ. Overall, it therefore appears difficult to reduce the intrinsically high p-type carrier concentration in this materials system.

## Conclusions

We have investigated the thermoelectric properties of the high mobility metal phosphide CaCuP when alloyed with As and have attempted to control the intrinsic p-type doping in this materials system. It has proved difficult to control the hole concentration by varying nominal sample composition, but the outstanding electronic properties of CaCuP are maintained in the alloyed system.

## Supporting Information Available

Additional X-ray powder diffraction and scanning electron microscopy characterization (PDF)

## Notes

Raw data on which this research is based can be accessed via the University of St Andrews Data Repository (https://doi.org/10.17630/1ddefe3e-34e9-4319-93bf-2de49185d65e).
