# Supplementary material for: Alloying and Doping Control in the Layered Metal Phosphide Thermoelectric CaCuP
Source: ACS Appl Electron Mater. 2023 Sep 14;6(5):2879–88. doi: 10.1021/acsaelm.3c00828 (PMC11137819; doi:10.1021/acsaelm.3c00828)
Supplement: Supplementary file 1 — el3c00828_si_001.pdf [file el3c00828_si_001.pdf]

Supporting Information for

Alloying and doping control in the layered metal phosphide thermoelectric CaCuP

Robert J. Quinn[1], Rajan Biswas[2], and Jan-Willem G. Bos[2*]

1. *Institute of Chemical Sciences, School of Engineering and Physical Sciences, Heriot-Watt University, Edinburgh, EH14 4AS, UK*
2. *EaStCHEM School of Chemistry, University of St Andrews, North Haugh, St Andrews, KY16 9ST, UK*

Email: j.w.g.bos@st-andrews.ac.uk

X-ray powder diffraction analysis

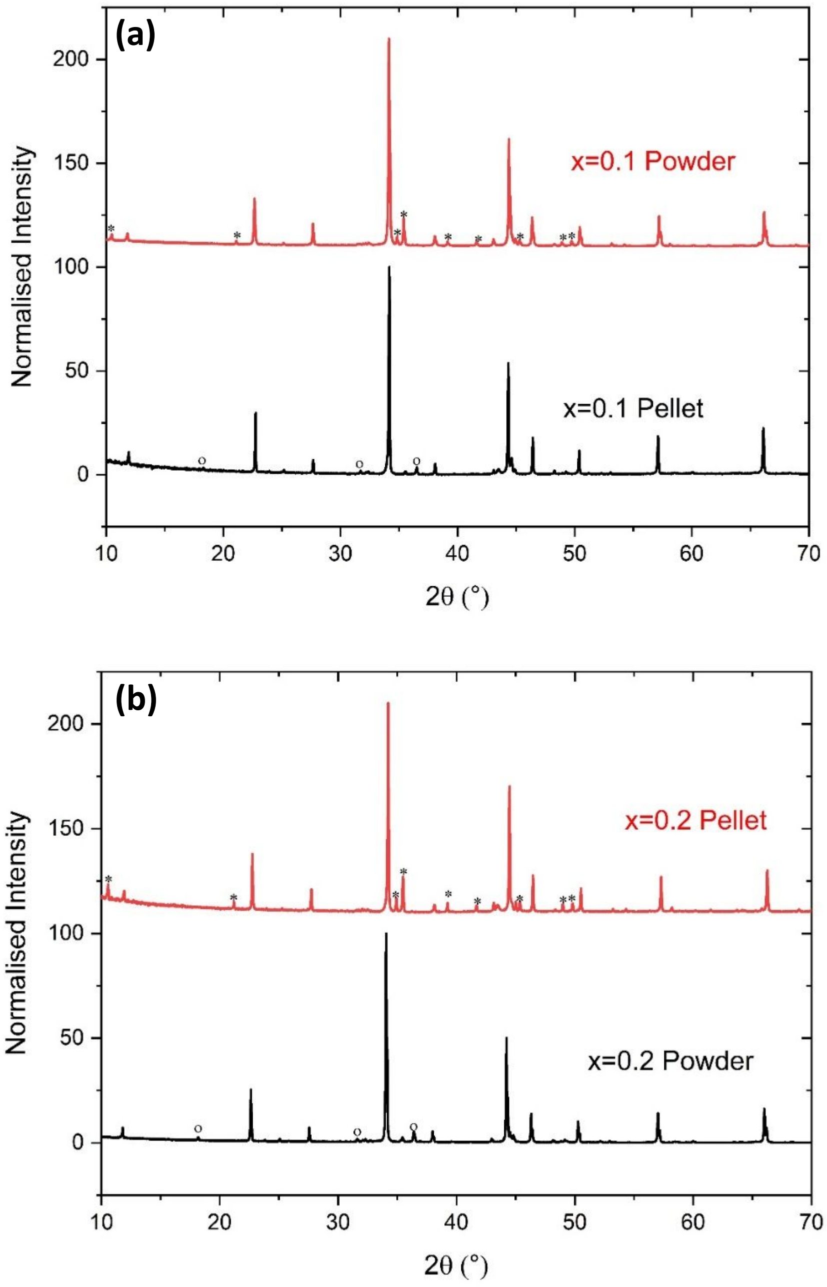

Fig. S1. Comparison of the X-ray diffraction patterns for the **(a)** x = 0.1 and **(b)** x = 0.2 $CaCuP_{1-x}As_x$ samples. Datasets were collected on finely ground powder (presented in the manuscript) and on a fragment of the same hot-pressed disk. The peaks marked (*) are nearly absent in the pellet for x = 0.1 (in the powder for x = 0.2). The peaks marked (°) show the opposite trend for both samples and are absent in the powder for x = 0.1 (and in the pellet for x = 0.2).

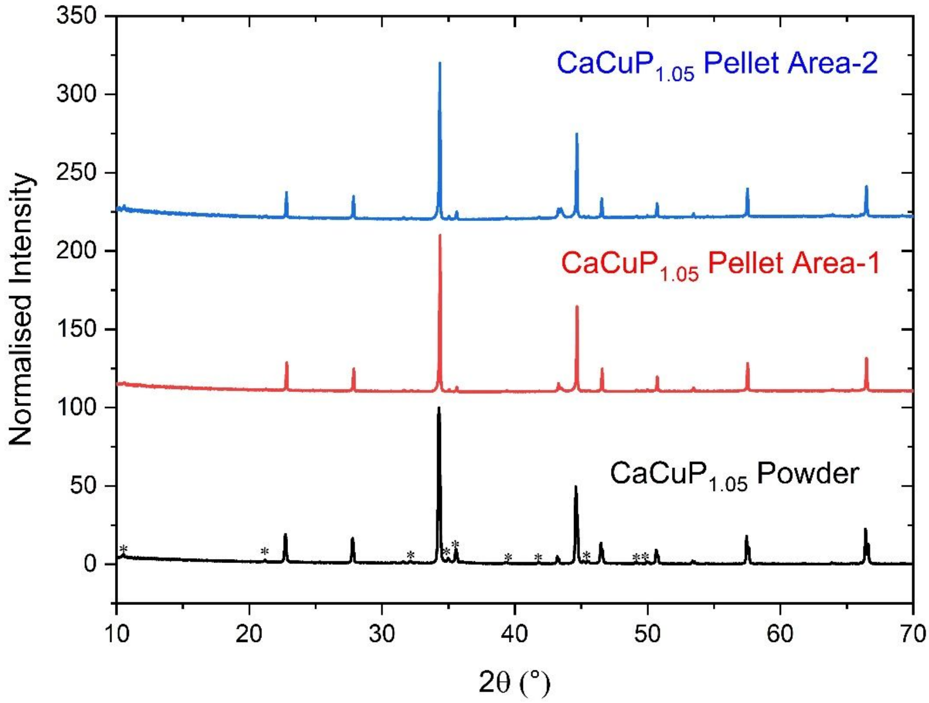

Fig. S2. Comparison of the X-ray diffraction patterns for the $CaCuP_{1.05}$ sample. Datasets were collected on finely ground powder (presented in the manuscript) and on two different fragments of the same hot-pressed disk. Similar trends are observed as in Fig. S1, with a varying degree of reduction of the (*) impurity phase in the pellet samples.

SEM-EDX analysis

Table S1. Elemental compositions for the $CaCuP_{1-x}As_x$ and phase boundary mapping CaCuP samples obtained from EDX

Sample	EDX Compositions (Ca:Cu:P:As) [at.%]	
	Bulk	Impurity
x = 0.1	27:33:37:3 (B-1)<br>32:34:30:4 (B-2)	16:54:28:2 (I-1)<br>16:53:28:3 (I-2)<br>17:15:16:2:O=50 (I-3)
x = 0.2	32:32:32:4 (B-1)	15:55:29:1 (I-1)<br>32:39:26:3 (I-2)
$CaCu_{1.05}P$	34:32:34:0 (B-1)	17:52:31:0 (I-1)<br>23:9:18:0:O=50 (I-2)
$Ca_{0.95}CuP$	32:36:32:0 (B-1)	25:38:37:0 (I-1)<br>20:0:15:0:O=65 (I-2)
$CaCuP_{1.05}$	34:31:35:0 (B-1)	30:33:37:00 (I-1)

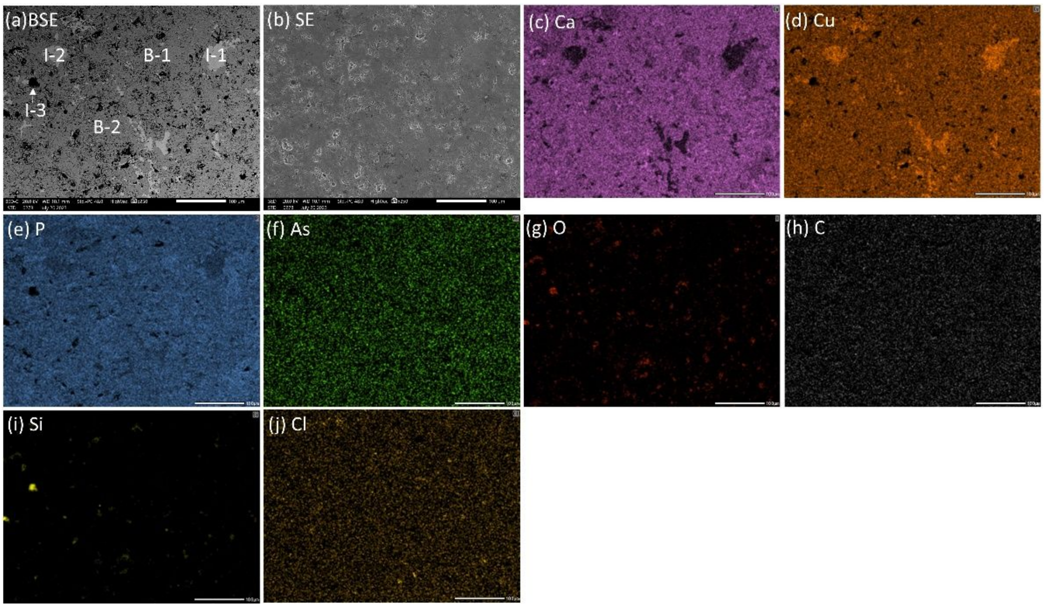

Fig. S3. Backscattered electron **(a)** and secondary electron **(b)** SEM images and elemental distribution maps **(c-j)** for the x = 0.1 $CaCuP_{1-x}As_x$ composition. Data collected on a polished surface of a hot-pressed disk. EDX compositions corresponding to the labelled positions are given in Table S1.

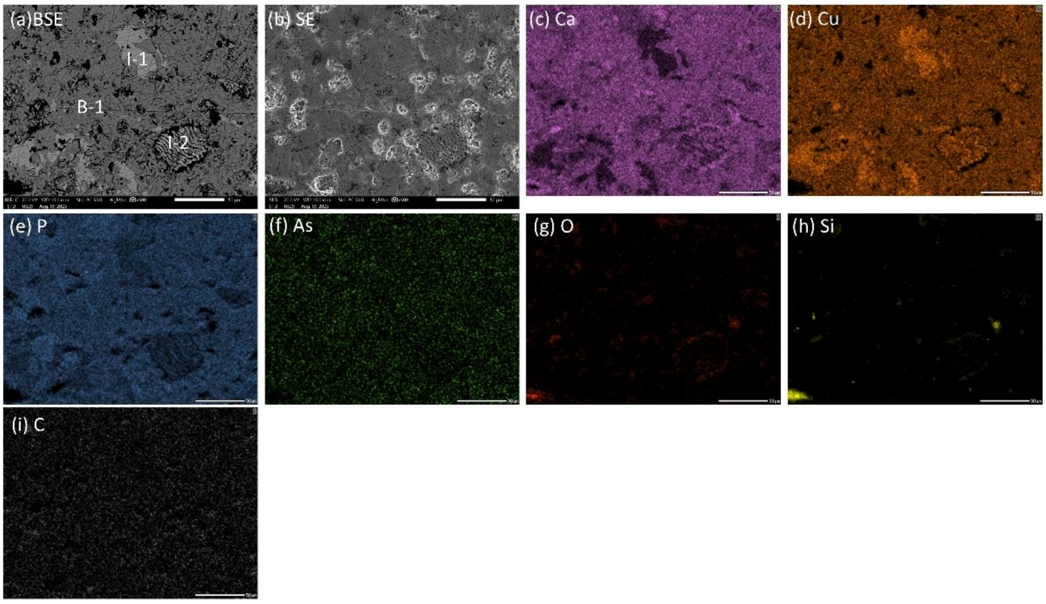

Fig. S4. Backscattered electron **(a)** and secondary electron **(b)** SEM images and elemental distribution maps **(c-i)** for the x = 0.2 $CaCuP_{1-x}As_x$ composition. Data collected on a polished surface of a hot-pressed disk. EDX compositions corresponding to the labelled positions are given in Table S1. I-2 is an example of a localised inclusion.

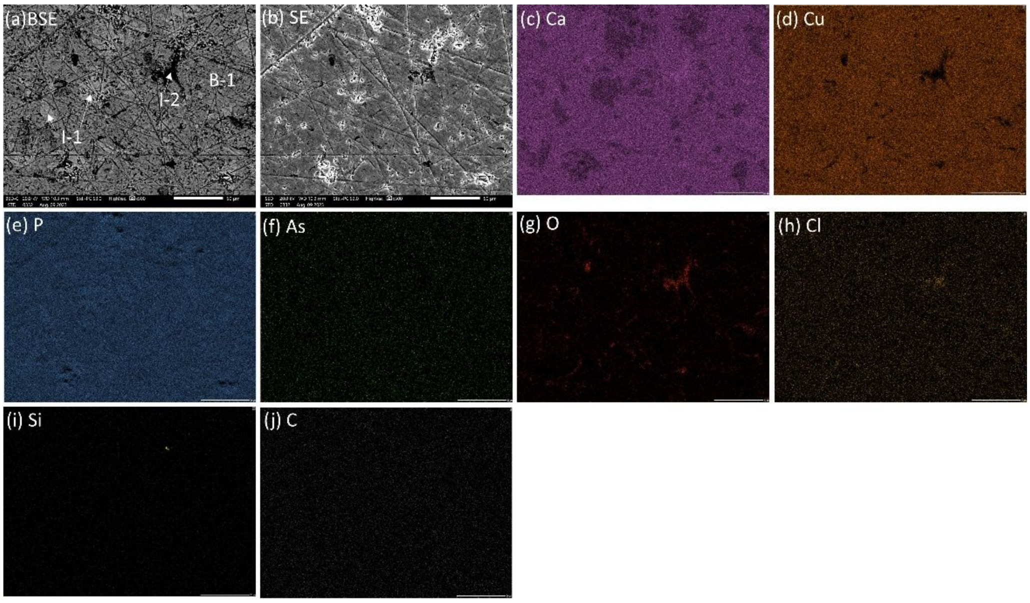

Fig. S5. Backscattered electron **(a)** and secondary electron **(b)** SEM images and elemental distribution maps **(c-j)** for the $Ca_{0.95}CuP$ composition. Data collected on a polished surface of a hot-pressed disk. EDX compositions corresponding to the labelled positions are given in Table S1. Dark region I-2 has increased oxygen concentration, possibly a phosphate phase.

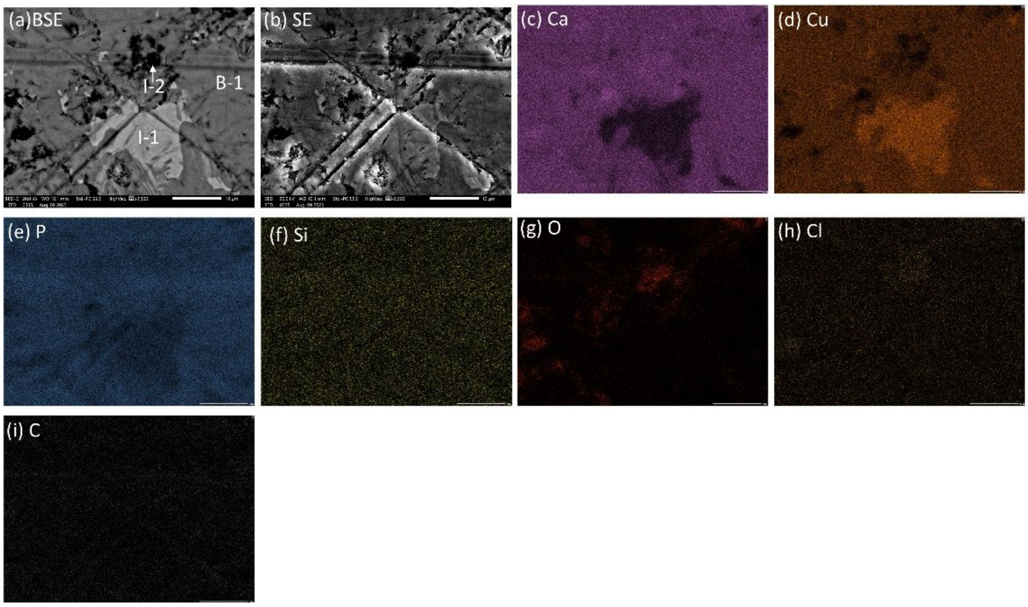

Fig. S6. Backscattered electron **(a)** and secondary electron **(b)** SEM images and elemental distribution maps **(c-i)** for the $CaCu_{1.05}P$ composition. Data collected on a polished surface of a hot-pressed disk. EDX compositions corresponding to the labelled positions are given in Table S1. Dark region I-2 has increased oxygen concentration, possibly a phosphate phase.

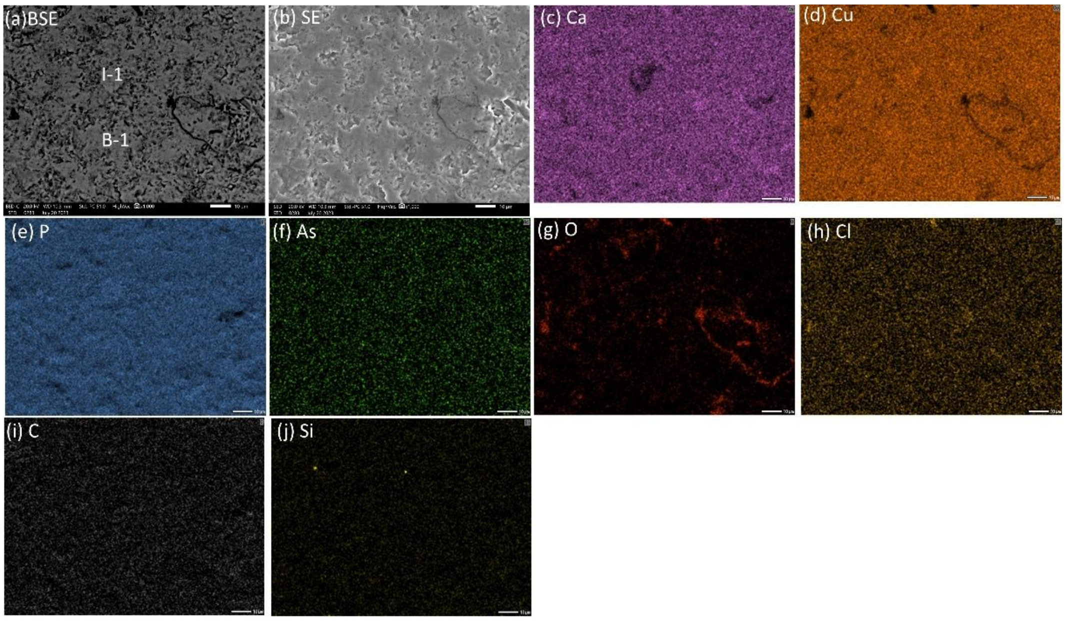

Fig. S7. Backscattered electron **(a)** and secondary electron **(b)** SEM images and elemental distribution maps **(c-j)** for the $CaCuP_{1.05}$ composition. Data collected on a polished surface of a hot-pressed disk. EDX compositions corresponding to the labelled positions are given in Table S1.
